# Brain Processing of Visual Information during Fast Eye Movements Maintains Motor Performance

**DOI:** 10.1371/journal.pone.0054641

**Published:** 2013-01-29

**Authors:** Muriel Panouillères, Valérie Gaveau, Camille Socasau, Christian Urquizar, Denis Pélisson

**Affiliations:** 1 Institut National de la Santé Et de la Recherche Médicale U1028, Centre National de la Recherche Scientifique UMR5292, Lyon Neuroscience Research Center, ImpAct Team, Lyon, France; 2 University Lyon 1, Lyon, France; University of Muenster, Germany

## Abstract

Movement accuracy depends crucially on the ability to detect errors while actions are being performed. When inaccuracies occur repeatedly, both an immediate motor correction and a progressive adaptation of the motor command can unfold. Of all the movements in the motor repertoire of humans, saccadic eye movements are the fastest. Due to the high speed of saccades, and to the impairment of visual perception during saccades, a phenomenon called “saccadic suppression”, it is widely believed that the adaptive mechanisms maintaining saccadic performance depend critically on visual error signals acquired *after* saccade completion. Here, we demonstrate that, contrary to this widespread view, saccadic adaptation can be based entirely on visual information presented *during* saccades. Our results show that visual error signals introduced during saccade execution–by shifting a visual target at saccade onset and blanking it at saccade offset–induce the same level of adaptation as error signals, presented for the same duration, but *after* saccade completion. In addition, they reveal that this processing of intra-saccadic visual information for adaptation depends critically on visual information presented during the deceleration phase, but not the acceleration phase, of the saccade. These findings demonstrate that the human central nervous system can use short intra-saccadic glimpses of visual information for motor adaptation, and they call for a reappraisal of current models of saccadic adaptation.

## Introduction

Vision is a crucial sense allowing human beings to interact with their environment. Humans produce from 150 000 to 200 000 saccadic eye movements daily, which underly fine vision. Saccades are fast and accurate conjugate ocular movements that bring the line of sight toward objects of interest. In some physiological (e.g. fatigue: [1], [2]) or pathological conditions (e.g. neuro-muscular lesion: [3]–[5]), oculomotor performance is impaired, threatening fine vision. When the accuracy of saccades is altered and the eyes repeatedly miss their goal, two complementary responses can unfold. The first one is an immediate response, achieved by a corrective movement that acquires the goal of the initial action [6]–[8]. The second one is a progressive response, consisting of an adaptation of the motor commands that results in a gradual recovery of the accuracy of subsequent movements [9]–[13]. Both responses require the detection of an error and here, we were interested in studying the visual error responsible for the second response, i.e. saccadic adaptation. Under laboratory conditions, visual error leading to saccadic adaptation is induced by systematically shifting the visual target during the execution of saccades, mimicking the visual consequence of an inaccurate movement [14]. When this intra-saccadic perturbation occurs repeatedly, the motor command of the saccade is gradually adapted by the brain, so that the eyes land progressively closer to the displaced goal (for reviews, see: [9]–[13]). In past research, the target is classically shifted at saccade onset, and it remains visible for a long duration (∼1 s) after saccade termination. Subjects are usually unaware of the target shift due to the “saccadic suppression of image displacement” phenomenon [15]. Because of this alteration of visual processing during saccades and of the high speed of the saccade, it is widely believed that error signals necessary for saccadic adaptation are acquired after saccade termination (e.g.: [16]–[21]; for reviews, see [9], [11]). However, it has never been explored whether intra-saccadic visual error, namely an error presented only during the saccadic response, can be used by the brain to adjust motor performance through adaptation.

The aim of this study was to test if intra-saccadic visual information is used to maintain saccade performance through adaptation. To do so, we limited the duration of the shifted target to a value shorter than the saccade duration (∼30 ms) and manipulated the occurrence and timing of the target shift. We compared two sessions in which adaptation was induced with an error presented either during the saccade (intra-saccadic session) or after the saccade completion (post-saccadic session). According to the widespread view that saccadic adaptation is induced based on post-saccadic visual information, we expected to observe larger saccadic adaptation in the latter session than in the former one. In two separate experiments, we tested this prediction for the two opposite adaptive changes of saccade amplitude, shortening and lengthening, which are known to rely on different mechanisms (for recent articles, see: [22]–[30]). In a third experiment, we evaluated whether the saccade acceleration or deceleration period was the most critical for intra-saccadic processing.

## Results

**Figure 1:**
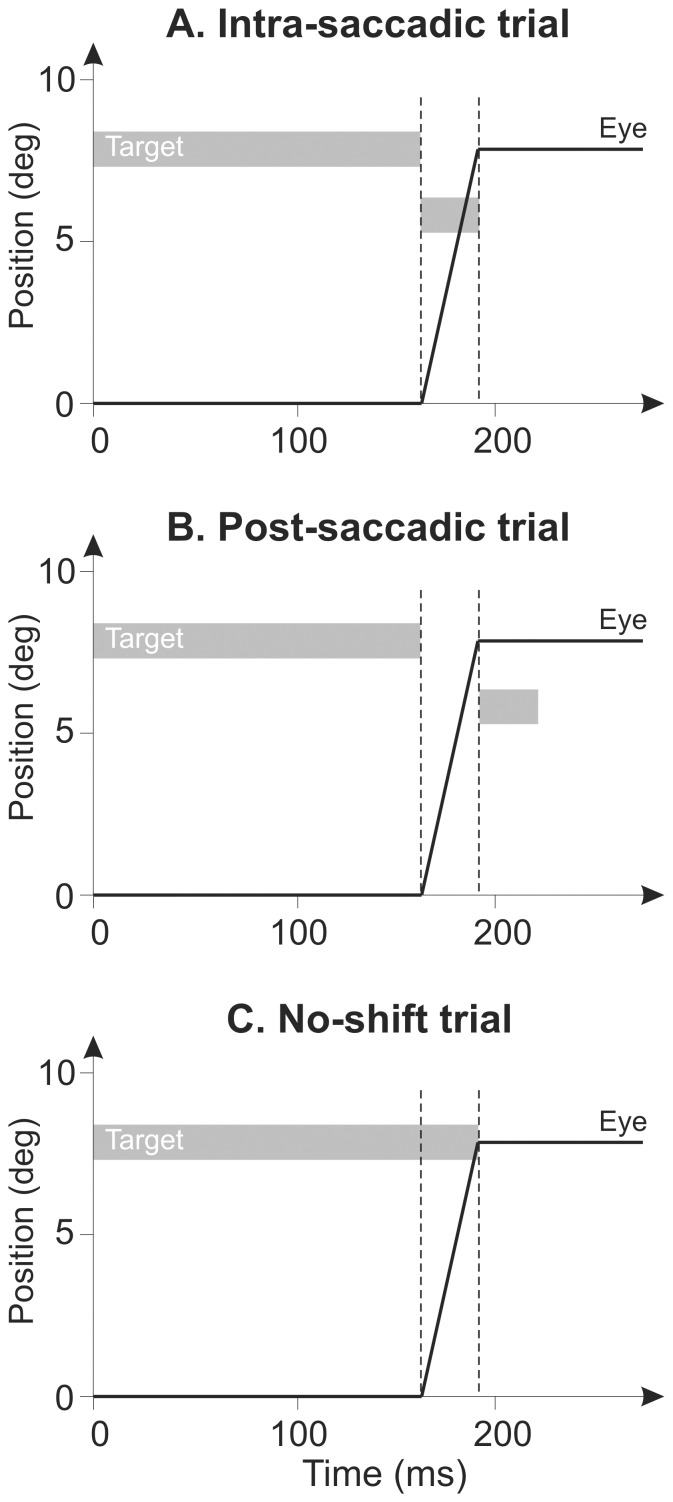
Exposure phase trials in the three sessions of the backward experiment. Schematics of backward adaptation trials for the intra-saccadic (A), post-saccadic (B) and no -shift (C) sessions are represented with eye (black line) and target (gray bars) positions as a function of time. Saccade onset and termination are symbolised with the vertical dashed lines.

In the first experiment, the direction of the target shift was backward, i.e. opposite to that of the saccade, in order to elicit an adaptive shortening of saccades. This backward experiment was performed by ten subjects and involved, in three separate sessions, two modified versions of the shifting target paradigm and one control paradigm without a target shift. Each of these three sessions comprised three phases (pre-exposure, exposure and post-exposure). In the exposure phase of the *intra-saccadic* session (Figure 1A), the visual error (target shift) was introduced at saccade onset and suppressed at saccade completion (target off); this allowed us to test the hypothesis that a strictly *intra*-saccadic visual error signal can trigger saccadic adaptation. In the exposure phase of the “post-saccadic” session (Figure 1B), the visual error was introduced *after* saccade completion, by shifting the target when the eyes landed, and this error remained available for the same duration as in the intra-saccadic session. In the exposure phase of the “no-shift” control session (Figure 1C), no visual error was introduced: the target remained at its initial location for the saccade duration and was turned off at saccade completion. In each of the three sessions, subjects performed four blocks of 48 exposure trials. In all trials, saccadic gain (ratio between saccade amplitude and target eccentricity saccade) was used to quantify saccadic accuracy.

**Figure 2:**
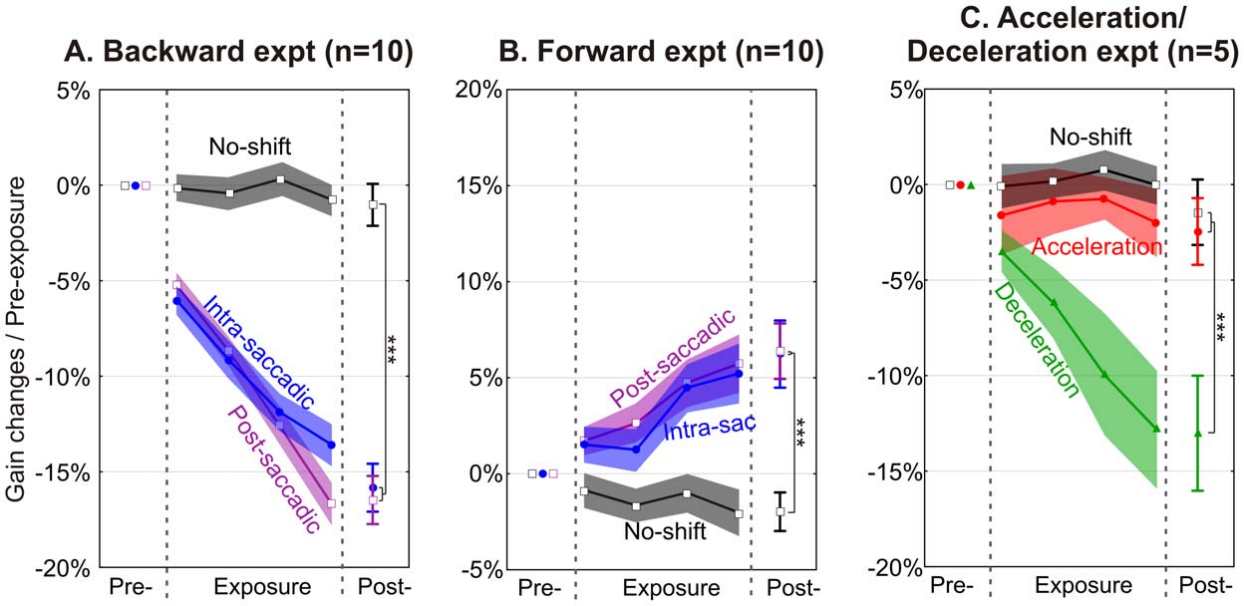
Adaptation development and after-effects in the three experiments. Gain changes computed relative to pre-exposure are represented as a function of the phases (pre-, four blocks of exposure, post-). For the backward (A) and forward (B) experiments the intra-saccadic (blue), post-saccadic (purple) and no-shift (black) sessions are shown separately. For the acceleration/deceleration (C) experiment, the acceleration (red), deceleration (green) and no-shift (black) sessions are represented separately. The symbols represent mean gain changes for pre- and post-exposure phases as well as for each block of the exposure phase. The shaded areas in the exposure phase represent one standard error of the mean (SEM). The error bars in post-exposure phase show SEMs. The asterisks indicate significant differences in after-effect (computed as post- versus pre- difference) between both the intra-saccadic and post-saccadic sessions with the no-shift session: *** *P*<0.001 (post-hoc Bonferroni tests). Note that the baseline gain of saccades measured during the pre-exposure phase was submitted for each experiment to a two-way ANOVA with session and saccade direction (rightward vs leftward) as factors. No main effect and no interaction was detected for any of the three experiments (backward: F_2,18_<0.71, *P*>0.5; forward: F_2,18_<1.28, *P*>0.3; acceleration/deceleration: F_2,8_<3.76, *P*>0.07). This indicates that baseline gain was similar for the three sessions of each experiment. Moreover, because of the lack of effect of the saccade direction factor, data of the rightward and leftward saccades were pooled for this figure and for subsequent analyses.

All three sessions began with a similar pre-exposure phase, aimed to measure the baseline gain of saccades directed toward a visual target disappearing at saccade onset. We first checked that the baseline gain did not differ between sessions (F_2,18_ = 0.71, *P = *0.50). The pre-exposure phase was then followed by the exposure phase. As shown in Figure 2A, a progressive decrease of saccade gain relative to the pre-exposure baseline occurred during the exposure phase of the intra-saccadic and post-saccadic sessions, but not during the no-shift session. This observation was confirmed by a two-way ANOVA testing the effects of the two factors, session and trial block, on the change of saccade gain relative to pre-exposure baseline. A significant effect of each factor (F_2,38_ = 60, *P*<0.001; F_5,95_ = 117, *P*<0.001 respectively) and a significant interaction were found (F_10,190 = _30, *P*<0.001). Post-hoc comparisons performed using Bonferroni tests revealed that in the no-shift session, saccade gain did not change during the exposure phase relative to the baseline (*P* = 1). Conversely, in the intra- and post-saccadic sessions, saccade gain progressively decreased, and significantly differed from baseline for the entire duration of the exposure phase (*P*<0.001). Moreover, gain changes did not differ between the intra-saccadic and post-saccadic sessions (*P*>0.10), although we note a trend for larger changes in the last block of the exposure phase in the post-saccadic session compared to the intra-saccadic session (P = 0.10). These results indicate that visual error information provided during the execution phase of saccades leads to a progressive decrease of saccade gain that reaches, after the 192 trials of the exposure phase, a level which is as large as that induced when the same information is provided after the eyes stopped moving.

Each session ended with a post-exposure phase in which, identical to the pre-exposure phase, the target disappeared at saccade onset. The change in saccadic gain between the post- and pre-exposure phases, in percent, was used to quantify the “adaptation after-effect”, a measure of the retention of adaptation [31]. As expected, no significant after-effect was observed in the no-shift session (Figure 2A, post-hoc Bonferroni tests, *P* = 1). In contrast, large and statistically significant after-effects were observed in the intra-saccadic (15.8±1.3%) and post-saccadic (16.5±1.3%) sessions (*P*<0.001) and these effects did not differ statistically between the two sessions (*P* = 1). In sum, for both intra-saccadic and post-saccadic sessions, the changes in saccade gain at the end of the exposure phase persisted during the post-exposure phase. This observation indicates that in both sessions, gain changes observed during the exposure phase resulted from adaptive mechanisms and not from strategic responses to the target shifts.

It is well-known that adaptive shortening and lengthening of saccades induced respectively by backward and forward target shifts, i.e. in the opposite or same direction as the saccade, rely on different mechanisms (for recent articles, see: [22]–[30]). Thus, we tested in a second experiment whether the findings reported above could be duplicated for adaptive saccade lengthening. Except for the fact that the target was shifted in the same direction as the saccade, this “forward experiment” was identical to the backward experiment, and involved ten other subjects. The results of this experiment (Figure 2B) were similar to those of the backward experiment, except of course for the sign of saccade gain changes. First, the baseline gain in this second experiment did not differ between sessions (F_2,18_ = 1.28, *P = *0.30). Second, for the intra-saccadic and post-saccadic sessions, saccade gain increased during the exposure phase relative to the baseline, mainly for the second half of this phase (two-way ANOVA revealing a significant main effect of block and session factors and a significant interaction between these factors: F_10,190_>9.4, *P*<0.001), and this increase was similar for the two sessions (post-hoc Bonferroni tests, *P* = 1). Finally, the adaptation after-effects did not differ between the intra-saccadic and the post-saccadic sessions (6.2±1.7% and 6.4±1.4%, respectively; post-hoc Bonferroni tests, *P* = 1), and in both cases revealed a significant difference of gain from baseline performance (*P*<0.001). It is worth pointing out that these after-effects (∼6%) were smaller than those measured in the backward experiment (∼16%), which is consistent with the well-known difference between forward and backward adaptation [22]–[30], [32]–[40]. The results of this second experiment on forward adaptation indicate that intra-saccadic visual errors are processed as effectively as post-saccadic ones, and thus confirm the findings obtained in the first experiment on backward adaptation.

Our finding that intra-saccadic visual information can induce strong adaptive changes raises the question of whether this information is processed during the acceleration or deceleration phase of saccades. To answer this question, a subset of 5 subjects from the backward experiment performed a third experiment comprising two sessions. In the “acceleration” session, the target was displaced backward at saccade onset and remained visible for only 10 ms during the saccade acceleration phase. In the “deceleration” session, the target remained at its initial location during the acceleration phase, was shifted backward after the velocity peak was reached (deceleration phase) and was blanked 10 ms later. The data of these two sessions were compared to the data of the no-shift session that these subjects had already performed in the backward experiment. As in the first two experiments, the baseline gain did not differ between the three sessions (F_2,8_ = 3.76, *P = *0.07). As shown in Figure 2C, the saccade gain during the exposure phase varied differently in the deceleration session as compared to the acceleration and no-shift sessions (two-way ANOVA, session × block interaction: F_10,90_ = 7.6, *P*<0.001). Indeed, a significant decrease in saccade gain relative to the baseline was observed only for the deceleration session (post-hoc Bonferroni tests, *P*<0.01), not for the acceleration and no-shift sessions (*P* = 1). We then compared gain changes during the deceleration session to gain changes measured in the same subgroup of subjects during the intra-saccadic session of the backward experiment, using a two-way (session and block) ANOVA. No difference in adaptation between these two sessions was found (session factor: F_1,0_ = 0.11, *P* = 0.75; session×block interaction: F_5,45_ = 0.24, *P* = 0.94). In the deceleration session, we also measured the instantaneous eye velocity at the time the target was turned off (130±48°/sec on average). A correlation analysis in our five subjects revealed that this eye velocity measure did not correlate with the level of gain changes in the post-exposure phase (R^2^ = 0.19, *P* = 0.20). This result suggests that the adaptive changes that develop during the deceleration session did not depend on the eye velocity at the time of target blanking. In conclusion, motor adaptation induced by visual information presented for only 10 ms during saccade deceleration was the same as adaptation induced by visual information presented for the entire saccade duration. In contrast, the same visual error, when presented during saccade acceleration, did not elicit any adaptation. Together, these results demonstrate that the deceleration phase is a critical period of saccade execution during which the presence of visual error signals can induce saccadic adaptation.

## Discussion

The results of this study provide evidence that the central nervous system can extract critically important information for motor control from visual stimuli that are presented exclusively during the flight time of a saccade. To produce an *immediate correction*, the brain must detect and process error signals as early as possible. Consistent with this view, it has been shown that intra-saccadic visual information can lead to immediate corrections (online modifications of large saccades and hand movements trajectory [41], [42], corrective saccades [43]–[45]). In contrast, in the case of adaptation mechanisms, the use of visual error signals has no consequence until the same motor response is reproduced, and we thus assumed that the early detection of error signals is not critical in this situation. Here, contrary to this expectation, we demonstrate for the first time that, in order to adapt saccades to environmental changes, the detection of visual information by the brain is not postponed to *after* the end of saccades. Even more surprisingly, large adaptive effects were observed when visual error information was presented for only 10 ms during the deceleration phase of saccades. Because the eyes are still travelling at high speed during saccade deceleration, these findings illustrate a remarkable capacity of the brain to process visual errors presented during saccades. We will now discuss possible mechanisms underlying this capacity of encoding target location during and after saccades and the implication of these findings for saccadic adaptation.

We propose two possible explanations of how visual errors are processed for saccadic adaptation. The first one is that different processing mechanisms are involved when the visual perturbation occurs either during the saccade or after the saccade. In the case where the stepped target is visible for a long duration (∼1 s) after saccade termination, it has been proposed that the error signals result from a comparison between the expected post-saccadic feedback, which is predicted based on a copy of the motor command (efference copy), and the actual feedback, which is sampled after saccade completion [16], [17], [46]. Both predicted and actual feedback signals result from egocentric coding of target relative to the subject’ gaze. The same hypothesis of egocentric coding of target position would also apply to the post-saccadic sessions of the current study. In contrast, for the intra-saccadic sessions, the central nervous system could favour target motion/displacement information over target position information because the former is conceivably less affected by the rapid sweeping of the target image across the retina. In this case, adaptation would not result from processing of egocentric signals of target position but rather from processing of allocentric information of the target’s intra-saccadic displacement. Allocentric coding refers to computations, which do not depend on the subject’s position but rather on visual landmarks present in the background. In our study where experiments were performed in complete darkness, we hypothesize that allocentric coding could use the initial target position as a landmark. Thus, whenever the initial and final target positions are nearly simultaneous (target step), this allocentric processing can provide a direct measure of the target step size and would be suitable to yield an accurate error signal independently of the speed of on-going eye displacement. However, if visual errors presented during or after the saccade are processed by two different mechanisms, different amounts of adaptation and of retention could have been expected between, on the one hand, the intra-saccadic and deceleration sessions and, on the other hand, the post-saccadic session. Our data do not agree with these predictions. Thus, the second and most parsimonious interpretation of our results is that the visual error is coded similarly whether presented during the saccade or after saccade termination. As already discussed above, given that the central nervous system encodes the egocentric position of the displaced target in the post-saccadic session, this second hypothesis proposes that the same egocentric coding strategy would take place during the intra-saccadic session. This egocentric coding would require the use of an efference copy signal that accurately reflects the instantaneous eye position during the on-going saccade. Previous studies found that a visual probe flashed during a saccade was systematically mislocalized, as revealed both by perceptual responses and by corrective saccadic responses [47]–[49]. These localization errors were taken as evidence that the ocular efference copy used in these tasks does not accurately code the dynamics of ocular displacement during a saccade. This suggestion is compatible with the fact that at the neurophysiological level, the temporal accuracy of dynamic eye position signals is still questioned (see for review: [50]). Note however, that in the aforementioned localization studies [47]–[49], very few corrective saccades were produced when the probe was close to the saccadic target (about 2–3°), a situation similar to that of the present study. Hence, there is very little evidence for oculomotor mislocalization in conditions that correspond to our task. Moreover, except in the study by Dassonville et al [47], the oculomotor mislocalization was associated with a similar mislocalization in a perceptual task which was performed simultaneously and which may have biased the motor response. Finally, and contrary to the previously mentioned studies, a pioneering study reported accurate oculomotor localization of a visual probe flashed during a saccade [44]. Thus, it is not completely settled if the central nervous system has access to an accurate eye position during saccade (perceptual versus oculomotor response, eccentricity of the target flash, etc…). Regarding the oculomotor system, our current results would then suggest a very accurate monitoring of on-line eye position during the saccade, allowing for an accurate coding of the target position for saccadic adaptation. Future studies will be necessary to describe the neural underpinning of this capacity to encode a visual error presented during the saccade.

Regardless of the allocentric and egocentric hypotheses presented above, how can we explain the lack of adaptation in the acceleration session? In this session, the target perturbation occurred at saccade onset, then the stepped target was switched off after 10 ms. Because saccadic suppression is known to peak near saccade onset and is almost absent at saccade offset (for review see: [51]), this phenomenon may reduce the visibility of targets presented in the acceleration phase. However, it has been shown previously that saccadic suppression of displacement was minimal for high luminance targets [52], such as the ones used in the present study. Thus the lack of adaptation in the acceleration session does not seem to be linked with saccadic suppression. More likely, the acceleration phase of the saccade may not be the critical time where the visual information is processed for saccadic adaptation.

The present study provides new insight into the mechanisms of sensorimotor adaptation. First, although previous studies have shown several differences between backward and forward adaptive mechanisms (for recent articles, see: [22]–[30]), we found that intra-saccadic visual information was processed in both forms of adaptation. Thus, intra-saccadic information processing appears to be a general mechanism involved in both adaptive lengthening and shortening of saccades. Second, the processing of intra-saccadic errors leads to the same adaptation level as that of visual errors presented to the stationary eyes after saccade termination. A previous study [19] has tested the effect of the post-saccadic duration of the visual target on the backward adaptation of reactive saccades. Similar to the present intra-saccadic session, the target was shifted at saccade onset but remained visible for 15, 50, 100 or 800 ms during the post-saccadic period (in 4 separate sessions). It was found that the shortest duration (15 ms) was sufficient to yield an optimal adaptation level (17.3±1.0%), i.e. which did not further increase with longer target durations (50, 100 and 800 ms). Note that the adaptation level found in the present backward experiment, regardless of whether the shifted target was presented during or after the saccade (15.8±1.3% and 16.5±1.3%, respectively), was similar to this optimal level. It is important to mention that in the present post-saccadic session, delaying the occurrence of the stepped target by about 30 ms (i.e. until the saccade termination), did not appear to impact saccadic adaptation. This is consistent with a previous study that reported a reduced amount of adaptation only when the occurrence of the target perturbation was delayed by more than about 100 ms after saccade offset [53]. Surprisingly, the presentation of the shifted target for only 10 ms during the saccade deceleration phase was sufficient to elicit a statistically similar level of adaptation as in the intra-saccadic and post-saccadic sessions. Taken together, these observations indicate that no further increase of saccadic adaptation can be elicited by extending the duration of visual error presentation outside the saccade deceleration period, whether during the saccade execution or even after the saccade termination. Therefore, these findings call for a reappraisal of current saccadic-adaptation models, which propose that visual error signals are sampled only after saccade completion (e.g. [16]–[21], see for reviews [9], [11]). Further studies are necessary to better understand these error processing mechanisms, for example by testing how error signals provided during the saccade interact with those presented after the saccade.

Overall, our findings clearly reveal that the brain can process visual information on-the-fly during visual saccades and that this intra-saccadic visual information plays a crucial role in motor control. An important goal for future studies is to further investigate the complex mechanisms underlying the processing of intra-saccadic visual information.

## Materials and Methods

### Subjects

Ten volunteers took part to the backward experiment (four males and six females, mean age: 23.0±2.7 years) and ten other subjects participated to the forward experiment (four males and six females, mean age: 23.9±6.4 years). A subset of five subjects from the backward experiment (5 females mean age: 22.8±1.9 years) also performed the acceleration/deceleration experiment. In total, sixteen subjects were naïve to the purpose of the study. All subjects had normal or corrected-to-normal vision.

### Ethics Statement

The study conformed with the Code of Ethics of the World Medical Association (Declaration of Helsinki) and was approved by the local ethics committee of the Lyon Neuroscience Research Center (INSERM U1028 - CNRS UMR 5292). The local ethics committee considered that a verbal consent was appropriate for the present behavioral study. Before the first experimental session of each subject, the experimenter explained the task to the subject and noted the statement of informed consent on her laboratory notebook.

### Apparatus

The experiment took place in a completely dark room of the ImpAct team lab with the subjects seating 114 cm from a concave spherical board. Red light-emitting diodes (LEDs, diameter: 3 mm; luminance: 12 cd/m^2^; wavelength: 625 nm) located along the horizontal meridian of the board at 0°, +8° or −8° were used as central fixation point (central LED) and visual targets (peripheral LEDs). A chin rest and a forehead rest restrained head movements. The horizontal position of right eye was recorded with an infrared video eye tracker at 1000 Hz (Eyelink 1000, tower mount set-up, SR Research, Canada). Before each recording session, the eye tracker was calibrated by asking the subjects to successively fixate three LEDs: one located in the central position (0°) and two presented at ±12°. Custom real-time software was used for the on-line monitoring of eye movements, the recording of eye movements for off-line analysis and the control of visual stimuli based on the instantaneous eye velocity or acceleration signals.

### Experimental Design

Three experiments were performed in this study. The backward and forward experiments were composed of three sessions: the intra-saccadic session, the post-saccadic session and the no-shift session. The session order was counterbalanced between subjects with the intra-saccadic session always preceding the post-saccadic session, such that the target duration in the latter session could be determined as the individual mean value obtained in the former session. The acceleration/deceleration experiment was sub-divided into the acceleration session and the deceleration session. In all experiments, each session was divided in 3 phases: a pre-exposure phase, an exposure phase and a post-exposure phase.

Pre- and post-exposure phases were identical for all sessions and composed of 24 trials (12 trials in each direction randomly intermixed). Each trial of the pre- and post-exposure phases began with a central fixation presented for a random duration comprised between 500 and 1500 ms. A target was then illuminated at ±8° and the fixation point was simultaneously turned off. Subjects were required to perform a saccade toward the target as fast and accurately as possible. When the eyes reached a velocity of 70°/s, the target was turned off to suppress any visual feedback.

Exposure trials differed between sessions. For the intra-saccadic session (Figure 1A), the target was displaced at saccade onset and switched off at saccade termination. For the exposure trials of the post-saccadic session (Figure 1B), the target disappeared at saccade onset and was re-illuminated upon saccade completion at the same shifted location and for the same mean duration as in the intra-saccadic session. For the no-shift session (Figure 1C), the target remained at ±8° for the entire saccade duration and was switched off at the end of the eye movement. In the exposure phase of the acceleration session, the target stepped at saccade onset (velocity threshold: 70°/s) and was switched off 10 ms later. In this condition, intra-saccadic visual information was exclusively presented during saccade acceleration. Finally, for the deceleration session, the target remained at its location during the acceleration phase of the saccade and was displaced as soon as eye acceleration dropped below 5000°/s^2^. The shifted target was then switched off 10 ms later.

All trials lasted from 2000 to 3000 ms and each trial followed the previous one without a delay. The target perturbation in the exposure phase of the intra-saccadic, post-saccadic, acceleration and deceleration sessions represented 25% of initial target eccentricity in the first half of the exposure phase and 40% in the second half. In the backward and acceleration/deceleration experiments, the target was shifted toward the fixation point to induce a decrease of saccade amplitude and in the forward experiment, it was directed away from the fixation point to produce an increase of saccade amplitude. The stepped target duration in the intra-saccadic sessions was on average 32.5±2.7 ms for the backward experiment and 34.4±3.6 ms for the forward experiment. By design, the average durations of stepped target were the same in the post-saccadic sessions. For each subject, a delay of at least 5–7 days separated two consecutive sessions.

### Data Analysis

Eye movement data were analysed off-line using laboratory made software developed with Matlab v.7.1 (Mathworks, MA, U.S.A.). The position and the time of the beginning and end of the primary saccades directed toward the targets were detected using a velocity threshold of 50°/s.

For each primary horizontal saccade, saccadic gain was obtained as the ratio between horizontal saccade amplitude (difference between final and initial eye positions) and retinal error (difference between target position and saccade starting position). For each session, mean saccade gain was calculated, separately for rightward and leftward saccades, in pre- and post-exposure phases, and for the exposure phase that was subdivided in four blocks (expo1, expo2, expo3 and expo 4). Saccades contaminated with a blink, not correctly detected on-line or with a gain outside [mean ±3SE] were excluded from further analysis (representing 4.5±4% of total trials in the backward experiment, 3.2±3% of total trials in the forward experiment and 4.3±5% in the acceleration/deceleration experiment). Gain change of each saccade recorded during the exposure and post-exposure phases was calculated with respect to the mean gain of the corresponding pre-exposure phase, separately for the two-saccade directions. Gain changes representing a decrease of saccade amplitude have negative values, whereas gain changes representing an increase of saccade amplitude have positive values.

Statistical analyses were performed using Statistica 9 (Statsoft Inc., Tulsa, OK, USA). Saccade gain in the pre-exposure phase was submitted, separately for the backward and forward experiments to an ANOVA with two within-subject factors: session (intra-saccadic vs post-saccadic vs no-shift) and saccade direction (left vs right). Because the saccade direction factor was never significant (see Results), rightward and leftward saccades were pooled for the remaining analyses. Then, saccade gain changes were submitted, separately for the backward and forward experiment, to two-way ANOVAs with the factors: session (intra-saccadic vs post-saccadic vs no-shift) and blocks of trials (pre- vs expo1 vs expo2 vs expo3 vs expo4 vs post-). For the acceleration/deceleration experiment, the design of the two-way ANOVA was the following: session (acceleration vs deceleration vs no-shift) and blocks of trials (pre- vs expo1 vs expo2 vs expo3 vs expo4 vs post-). To compare the data of the deceleration session to the data of the intra-saccadic session collected in the same subset of five subjects, saccade gain changes was submitted to a final two-way ANOVA with the factors: session (intra-saccadic × deceleration) and blocks of trials (pre- vs expo1 vs expo2 vs expo3 vs expo4 vs post-). Significant ANOVAs were followed by post-hoc comparisons using Bonferroni tests. Significant threshold was set at *P*<0.05.
